# The Antiseptic Treatment of Typhoid Fever

**Published:** 1882-09

**Authors:** 


### THE ANTISEPTIC TREATMENT OF TYPHOID FEVER.

At a meeting of the Société Médicale des Hôpitaux, June 9th, M. Ferrand presented the candidate's thesis of Professor Desplat, of Lille, upon the comparative action of carbolic acid and salicylate of soda. The views presented were that the above drugs were excellent antipyretic and antialgesic agents—sure, rapid and permanent in their action, but, at the same time, easily eliminated, and, therefore, but slightly dangerous. Except in acute rheumatism, M. Desplats did not find any marked difference in their action.—*Medical Record.*

